# Improving Mobilization of Foreign DNA into Zymomonas mobilis Strain ZM4 by Removal of Multiple Restriction Systems

**DOI:** 10.1128/AEM.00808-21

**Published:** 2021-09-10

**Authors:** Piyush Behari Lal, Fritz Wells, Kevin S. Myers, Rajdeep Banerjee, Adam M. Guss, Patricia J. Kiley

**Affiliations:** a DOE Great Lakes Bioenergy Research Center, University of Wisconsin—Madison, Madison, Wisconsin, USA; b Department of Biomolecular Chemistry, University of Wisconsin—Madison, Madison, Wisconsin, USA; c Wisconsin Energy Institute, University of Wisconsin—Madison, Madison, Wisconsin, USA; d Biosciences Division, Oak Ridge National Laboratory, Oak Ridge, Tennessee, USA; e Center for Bioenergy Innovation, Oak Ridge National Laboratory, Oak Ridge, Tennessee, USA; North Carolina State University

**Keywords:** *Zymomonas mobilis* ZM4, restriction modification system, genome defense, conjugation efficiency of foreign genes, type I restriction enzymes

## Abstract

Zymomonas mobilis has emerged as a promising candidate for production of high-value bioproducts from plant biomass. However, a major limitation in equipping Z. mobilis with novel pathways to achieve this goal is restriction of heterologous DNA. Here, we characterized the contribution of several defense systems of Z. mobilis strain ZM4 to impeding heterologous gene transfer from an Escherichia coli donor. Bioinformatic analysis revealed that Z. mobilis ZM4 encodes a previously described *mrr*-like type IV restriction modification (RM) system, a type I-F CRISPR system, a chromosomal type I RM system (*hsdMS_c_*), and a previously uncharacterized type I RM system, located on an endogenous plasmid (*hsdRMS_p_*). The DNA recognition motif of HsdRMS_p_ was identified by comparing the methylated DNA sequence pattern of mutants lacking one or both of the *hsdMS_c_* and *hsdRMS_p_* systems to that of the parent strain. The conjugation efficiency of synthetic plasmids containing single or combinations of the HsdMS_c_ and HsdRMS_p_ recognition sites indicated that both systems are active and decrease uptake of foreign DNA. In contrast, deletions of *mrr* and *cas3* led to no detectable improvement in conjugation efficiency for the exogenous DNA tested. Thus, the suite of markerless restriction-negative strains that we constructed and the knowledge of this new restriction system and its DNA recognition motif provide the necessary platform to flexibly engineer the next generation of Z. mobilis strains for synthesis of valuable products.

**IMPORTANCE**Zymomonas mobilis is equipped with a number of traits that make it a desirable platform organism for metabolic engineering to produce valuable bioproducts. Engineering strains equipped with synthetic pathways for biosynthesis of new molecules requires integration of foreign genes. In this study, we developed an all-purpose strain, devoid of known host restriction systems and free of any antibiotic resistance markers, which dramatically improves the uptake efficiency of heterologous DNA into Z. mobilis ZM4. We also confirmed the role of a previously known restriction system as well as identifying a previously unknown type I RM system on an endogenous plasmid. Elimination of the barriers to DNA uptake as shown here will allow facile genetic engineering of Z. mobilis.

## INTRODUCTION

Zymomonas mobilis has several metabolic attributes that are advantageous for engineering strains to produce biofuels and other valuable commodities from lignocellulosic biomass on an industrial scale (1–4). However, genetic engineering of this organism has been challenging in part due to the presumed restriction of foreign DNA. To optimize the metabolism of Z. mobilis and unlock its full potential for industrial production of compounds of interest, introduction of foreign DNA needs to be more reliable and efficient. Recently, we developed a markerless genetic approach to add or remove genes from Z. mobilis strain ZM4 (5). Here, we used this method to delete genes encoding Z. mobilis restriction systems to test their effects on improving uptake of foreign DNA.

Bacteria have evolved several types of defense systems as barriers to invasion by foreign DNA. These activities usually comprise one or more of the four types of restriction modification (RM) systems or multiple types of CRISPR-Cas systems. The diversity of restriction systems in bacterial species and the challenges encountered in circumventing these systems to facilitate genetic engineering have been well documented (6–15). The type I RM systems are the most complex because the DNA sequence specificity determinant (HsdS), the DNA methyltransferase (HsdM), and the endonuclease (HsdR) are each encoded by separate genes and function as an oligomer. HsdS recognizes the methylation status of a specific bipartite DNA sequence motif. If the DNA motif is hemimethylated, then HsdM in complex with HsdS methylates the unmethylated DNA strand. If the DNA motif is unmethylated or incorrectly methylated, then HsdR cleaves the DNA, usually at a distance from the recognition sequence (6, 16). Type II RM systems also have methyltransferase and endonuclease activities but are quite diverse in their subunit composition and cleave DNA at or near their recognition sequence (12). Nevertheless, as with type I enzymes, base methylation of the target site results in protection from DNA cleavage by the cognate type II endonuclease. Type III RM systems consist of two proteins with endonuclease and DNA methylation activities, but these enzymes usually recognize short asymmetric sequences in an inverted repeat orientation (17, 18). Type IV RM systems contain only endonucleases, and restriction activity is directed against methylated invading DNA (19). These type IV enzymes are suggested to be promiscuous for their target sequence (20). CRISPR-Cas systems are quite diverse and composed of several proteins that defend against invading bacteriophages or plasmids (13, 14).

Because restriction of DNA hinders development of genetic systems in bacteria, several approaches have been exploited to evade RM systems. In some cases, propagating plasmid DNA in a methylation-deficient Escherichia coli strain (21) prior to transformation decreases restriction. An approach to evade type I RM systems is the electroporation of DNA mixed with a type I RM system inhibitor protein, OCR (6, 21, 22). This protein mimics B-form DNA, and binding to a type I restriction enzyme prevents the enzyme from binding to target DNA (23). Genetic engineering to remove type I RM target sequences from heterologous DNA of interest has also been deployed (8, 24). Expression of organism-specific methyltransferases in an E. coli plasmid-propagating strain to methylate heterologous DNA before introduction into relevant bacteria also shows promise (25–27). However, these targeted approaches sometimes do not mitigate restriction from other RM systems; methyltransferases may not express well in E. coli, or the approach may require prior knowledge of the host’s restriction systems to devise an effective strategy. Thus, these approaches may not confer immunity against all RM systems. When gene deletion technologies exist for a strain, an alternative approach is to delete genes involved in restriction.

In the case of Z. mobilis ZM4, the understanding of restriction systems is incomplete, making it challenging to develop an effective strategy to evade all defense systems of this organism. REBASE, a restriction enzyme database (28), provides one resource for the prediction of endogenous restriction enzymes in Z. mobilis ZM4. Independent disruptions of a gene predicted to encode the specificity determinant of a previously annotated type I RM system (*hsdS_c_*; ZMO1933) and a gene predicted to encode a type IV RM system (*mrr*; ZMO0028) (22, 25) resulted in small increases in DNA uptake efficiencies for exogenous plasmids, supporting the predicted roles of *mrr* and *hsdS_c_* in DNA restriction. A CRISPR-Cas system of Z. mobilis ZM4 was also recently characterized (7, 14).

In this study, we extend these earlier findings of Z. mobilis ZM4 RM systems to show that elimination of the previously annotated type I and IV RM systems is not sufficient to improve uptake of foreign DNA, indicating the presence of other restriction systems. Using a bioinformatic approach and high-throughput single-molecule real-time whole-genome methylome sequencing, we identified an additional type I RM system encoded on a native plasmid of Z. mobilis ZM4. This analysis also suggests that the previously annotated chromosomal type I RM system (HsdMS_c_) appears to have a nuclease domain fused to the HsdM subunit (29, 30). By creation of a series of strains with deletions of one or more restriction system genes, we found that removal of both the chromosomal type I RM system and the plasmid type I RM system was needed to maximally increase the uptake efficiency of foreign DNA. The availability of a suite of markerless strains with different combinations of RM systems eliminated provides more genetically tractable strains for metabolic engineering endeavors.

## RESULTS

### Bioinformatic predictions of RM systems of Z. mobilis ZM4.

**FIG 1:**
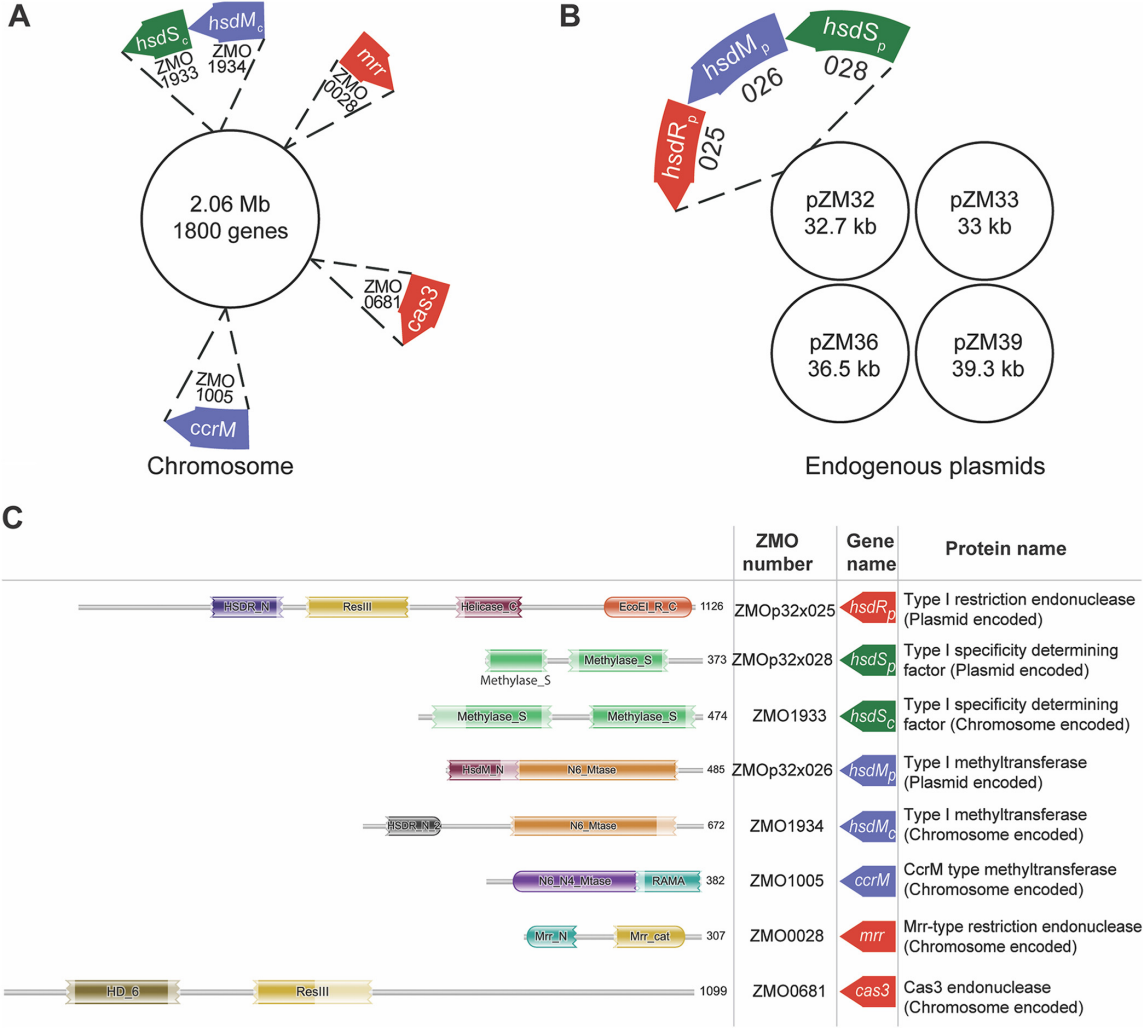
Gene location and sequence features of Z. mobilis ZM4 restriction systems. (A and B) Schematic representation showing the location of restriction system genes in the chromosome (A) and plasmids (B). Genes encoding endonucleases are shown in red, those for methyltransferases are in blue, and those for specificity-determining factors are in green. (C) Summary of RM system sequence features and matches with the Pfam database. The region of peptide sequence matching domains of Pfam clans are represented graphically. The protein superfamily clan of the domains for HsdR_p_ identified by the phmmer search tool, CDD domain search tool, and UniProt is cl36022, that for HsdS_c_ is cl38903, those for HsdS_p_ are cl35887 and cl38903, those for HsdM_c_ are cl29110 and cl37510, those for HsdM_p_ are cl37510 and cl13579, those for CcrM are c17173 and cl16759, those for Mrr is cl34341, and that for Cas3 is cl28317. In HsdM_c_, an N-terminal domain of HsdR that belongs to protein superfamily clan cl29110 is fused with HsdM. The jagged edge of a domain at the N or C terminus indicates that the sequence did not extend to the first or last position in the HMM database, respectively.

To develop strains with improved uptake efficiency of foreign DNA, we used bioinformatic predictions to identify genes encoding restriction systems in Z. mobilis ZM4 (31) to target for deletion (Fig. 1). As expected, the previously described ZMO0028 (*mrr*) (25) contains a domain typical of the Mrr superfamily of type IV restriction endonucleases, and ZMO0681 (*cas3*) (7, 14) contains a domain expected for type I-F CRISPR-Cas systems (13). ZMO1005 contains domains conserved in DNA methyltransferases and is 60% similar with the beta-group methyltransferase, CcrM of *Caulobacter* sp. (32). The presence of conserved motif IV and motif I of CcrM (33) in ZMO1005 indicates that ZMO1005 is a CcrM-like methyltransferase that is a housekeeping methyltransferase and is not typically associated with RM systems. The previously described ZMO1933 (*hsdS*) (25) has a domain belonging to the RM TypeI_S_TRD-CR-like superfamily, consistent with its encoding the specificity-determining factor of a type I RM system (30, 34). The neighboring gene ZMO1934 has a domain representative of the *N*^6^-methyltransferase superfamily, consistent with its proposed function as the methyltransferase component of a type I RM system along with specificity factor ZMO1933 (34). However, no protein with all of the domains expected for the HsdR subunit of a type I RM system was detected in our bioinformatic search. Rather, ZMO1934 had a conserved domain found in the N terminus of type I restriction endonuclease (HsdR_N), indicating a possible N-terminal fusion of a HsdR with HsdM. Such fusion proteins, which lack the motor and helicase domains of HsdR, have sometimes been reclassified to the quite diverse group of type II RM systems (6, 29).

We also found a previously unannotated type I RM system encoded on the native plasmid ZMOp32. Three adjacent genes (*zmop32x025* [*hsdR_p_*], *zmop32x026* [*hsdM_p_*], and *zmop32x028* [*hsdS_p_*]) were identified as having conserved domains of the HsdR superfamily, the i6-methyltransferase superfamily, and the RM TypeI_S_TRD-CR-like superfamily, respectively, suggesting that they encode three subunits of a type I RM system in Z. mobilis ZM4. The plasmid-encoded type I RM system appears to be distinct from the chromosomal system, since there is only 34% and 28% amino acid sequence identity between the two HsdSs and the two HsdM protein sequences, respectively. To differentiate between the subunits of the chromosome-encoded type I RM system and the plasmid-encoded type I RM system in Z. mobilis ZM4, we refer to the genes with a subscript “c” for the chromosomal type I RM system (*hsdMS_c_*) and “p” for the plasmid type I RM system (*hsdRMS_p_*).

### Phylogenetic analysis of type I RM systems in Z. mobilis strains.

**FIG 2:**
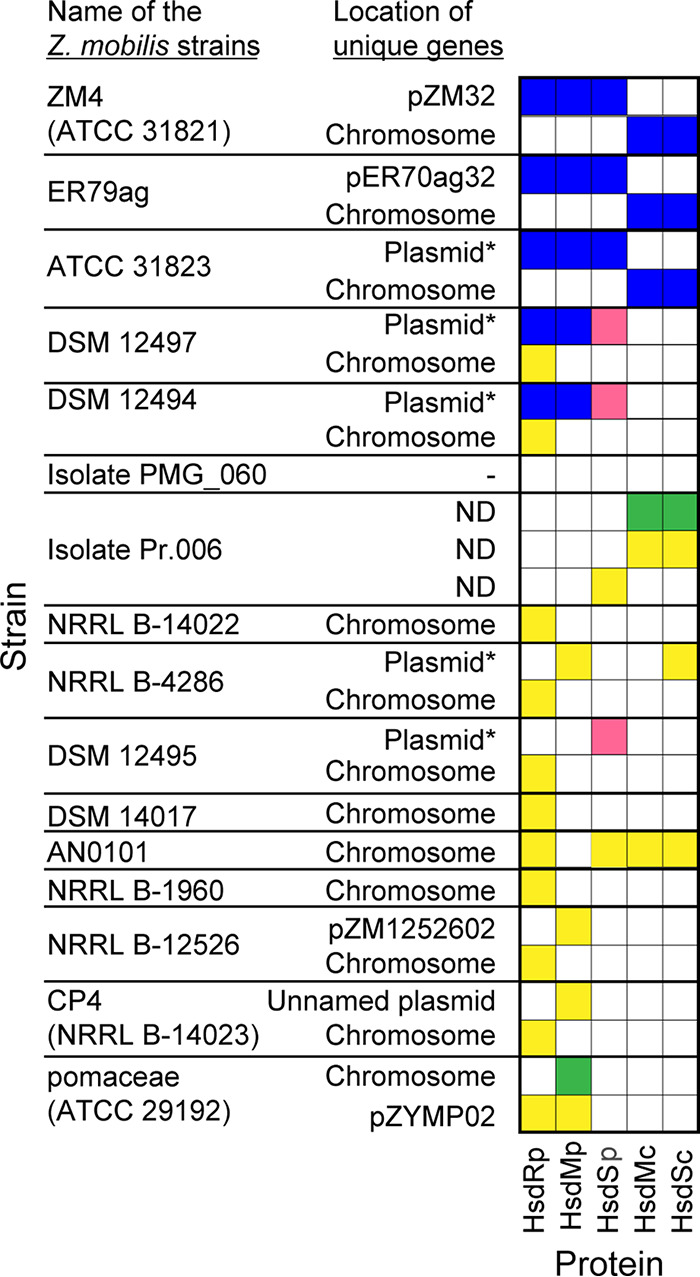
Amino acid sequence identity of Z. mobilis ZM4 HsdR_p_, HsdM_p_, HsdS_p_, HsdM_c_, and HsdS_c_ across 16 unique Z. mobilis strains. Blue, red, green, and yellow boxes indicate >96%, 41 to 50%, 31 to 40%, and 20 to 30% identity, respectively, to the indicated Z. mobilis ZM4 genes from the tBLASTn result. White boxes indicate that no homolog to the Z. mobilis ZM4 gene was detected in the tBLASTn result. If the genome was not complete, contigs greater than 2 Mb are indicated as chromosomal and the contigs smaller than 50 kb are indicated as plasmid* unless already specified as plasmid in the assembly. The sequence assembly of Z. mobilis isolate Pr.006 contained more than 700 contigs of 10 to 20 kb, and the location of matching regions was not determined (ND).

Since two type I RM systems were found in Z. mobilis ZM4 (31), we asked whether other Z. mobilis strains have the same systems. Using a local tBLASTn function, we searched the genome sequence of 16 Z. mobilis strains for sequences homologous to the HsdR_p_, HsdM_p_, HsdS_p_, HsdM_c_, and HsdS_c_ (Fig. 2). Two strains (ER79ag and ATC31823) contain the same complement of HsdMS_c_ and HsdRMS_p_ RM systems as strain ZM4, since proteins 100% identical to HsdRMS_p_ and HsdMS_c_ were identified from their genome sequences. Two other strains (DSM12497 and DSM12494) encode proteins identical to HsdRM_p_, but the ortholog of HsdS_c_ shares only 41% identity to Z. mobilis ZM4. The remaining strains appear to lack orthologs to either RM system, indicating that neither system is part of the core Z. mobilis genome.

### Methylome sequencing to identify type I RM system target sequences in Z. mobilis ZM4.

To identify the target sequences for the HsdMS_c_ and HsdRMS_p_ RM systems encoded on the chromosome and native plasmid pZM32, respectively, we compared the genomic methylation pattern of the parent strain to mutants lacking one or both Hsd systems. We reasoned that this would allow us to confirm the recognition sequence of HsdMS_c_ and identify the recognition sequence for HsdRMS_p_. Z. mobilis ZM4 strains lacking the specificity subunits of either the chromosomal (Δ*hsdS_c_*) or the chromosomal and plasmid Hsd systems (Δ*hsdS_c_* Δ*hsdS_p_*), which should eliminate both methylation and restriction activities, were constructed. Using single-molecule real-time DNA sequencing, we identified all methylated base modifications in the genome of the parent Z. mobilis ZM4 strain and compared this pattern with the methylome pattern of the mutant strains lacking one or both of the predicted Hsd systems. The results of the parent strain indicated methylation of adenine to *N*^6^-methyladenine at three different target sequences: 5′CAGN_4_CTG, 5′GAAGN_7_TCC, and 5′GANTC, where the underline represents adenine methylation (Table 1). The Δ*hsdS_c_* strain lacked adenine methylation of the bipartite sequence 5′ CAGN_4_CTG, confirming this sequence as the HsdS_c_ target site. The Δ*hsdS_c_ hsdS_p_* strain additionally lacked adenine methylation of 5′GAAGN_7_TCC, indicating that the latter sequence is the HsdS_p_ target site. These results also support the bioinformatic analysis that the two Hsd systems are genetically distinct and recognize distinct DNA sequences. GANTC is typically recognized by CcrM, which is presumably the case here as well.

**TABLE 1:** Methylome sequencing data

Genotype of strain	Methylated recognition sequence motif^*a*^	Total no. of methylated sequence motifs detected^*b*^	% of motifs methylated in whole genome^*c*^
Wild type	CAGN_4_CTG	938	100
	GAAGN_7_TCC	325	100
	GANTC	8,599	99.24
*hsdS_c_*	CAGN_4_CTG	0	0
	GAAGN_7_TCC	325	100
	GANTC	8,470	97.78
*hsdS_c_ hsdS_p_*	CAGN_4_CTG	0	0
	GAAGN_7_TCC	0	0
	GANTC	8,541	98.64

a Underlining indicates adenine methylation.

b Total number of methylated sequence motifs detected by PacBio SMRT DNA sequencing of genomic DNA (chromosomal and plasmids).

c Percentage of sequence motifs present in the genome of indicated strain which were methylated.

### Establishing the functional relevance of the type I RM systems.

**FIG 3:**
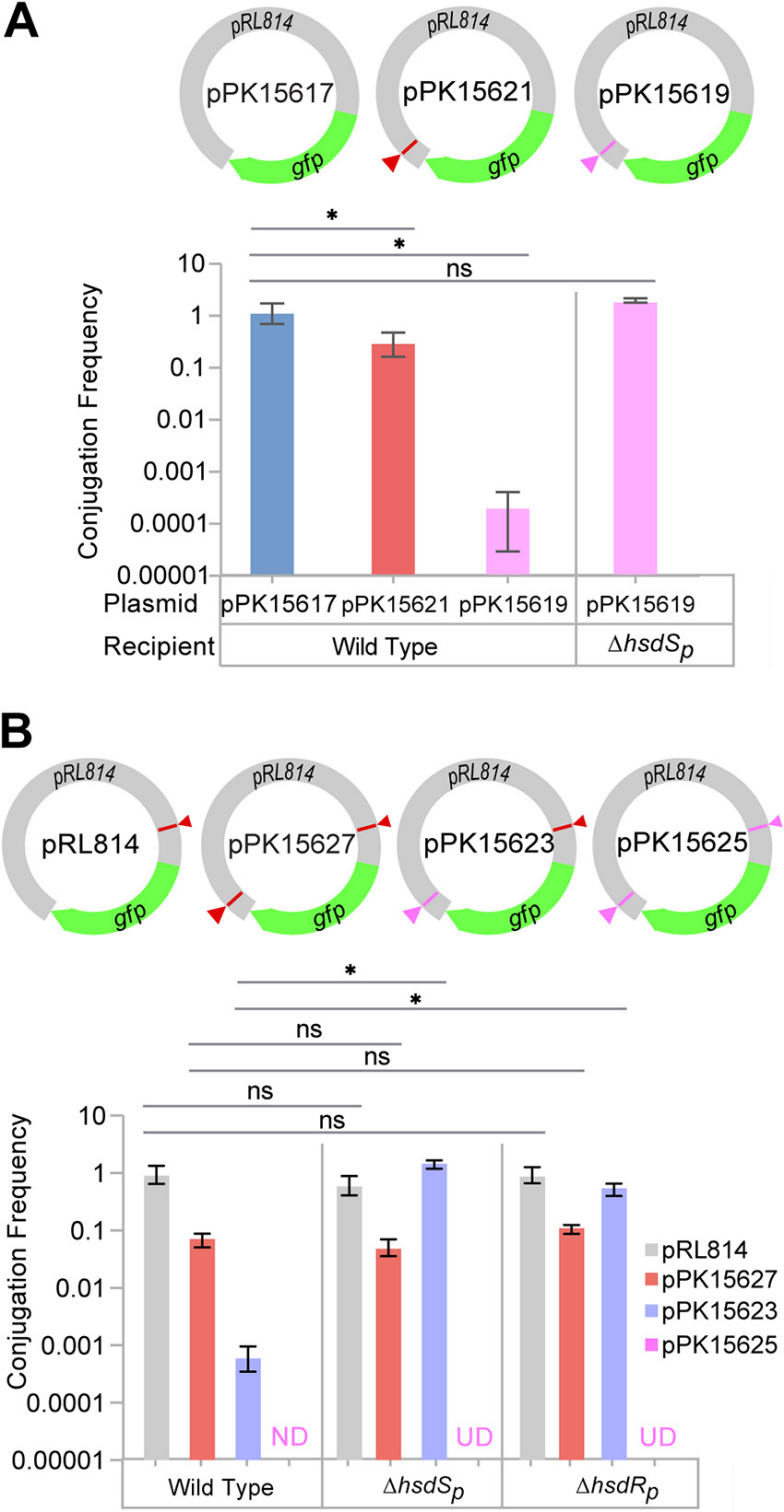
Functional relevance of the type I RM target sequences as measured by conjugation frequency. (A) The conjugation frequency of pPK15617 into Z. mobilis ZM4 (1.4 × 10^−5^ ± 0.5 × 10^−5^) served as a normalization factor for comparing the conjugation efficiency (*y* axis) of plasmids in wild-type and mutant ZM4 strains as indicated. (B) The conjugation frequency of pRL814 into Z. mobilis ZM4 (0.97 × 10^−3^ ± 0.5 × 10^−3^) served as a normalization factor for comparing the conjugation efficiency (*y* axis) of plasmids in wild-type and mutant ZM4 strains as indicated. When conjugation of a plasmid was below the limit of detection (0.00001), the sample is marked “ND” (not detected). When conjugation experiment of a plasmid was not done, it is marked “UD” (undetermined). Error bars represent the standard deviations of the conjugation frequency means obtained from three independent experiments. Statistical significance was determined using a paired Student's *t* test (*, *P* < 0.05; **, *P* ≤ 0.01; ***, *P* ≤ 0.001; ns, not significant). On the plasmids, red triangles represent restriction sites recognized by HsdS_c_ and pink triangles represent restriction sites recognized by HsdS_p_.

To determine if these systems impact Z. mobilis DNA uptake, we compared the conjugation efficiency of plasmids engineered to contain no, one, or two HsdS_c_ and HsdS_p_ synthetic target sites (Fig. 3). Compared to the plasmid lacking any HsdS_c_ and HsdS_p_ target sites (pPK15617), the conjugation efficiency of the plasmid containing one HsdS_c_ target site (5′CAGN_4_CTG; pPK15621) was reduced 5-fold in Z. mobilis ZM4 (Fig. 3A). The plasmid containing one HsdS_p_ target site (5′GAAGN_7_TCC; pPK15619) was more dramatically restricted, since the efficiency of conjugation into the parent strain was 4,900-fold less than that of the plasmid lacking any sites (pPK15617). Elimination of *hsdS_p_* restored the conjugation efficiency of pPK15619 to near that of the plasmid lacking any HsdS_c_ and HsdS_p_ target sites (pPK15617), indicating the specific role of the HsdRMS_p_ system in restriction of the GAAGN_7_TCC sequence present on plasmid pPK15619. These results also suggest that the HsdRMS_p_ restriction system is more active in Z. mobilis ZM4 than HsdMS_c_.

We also measured the impact of additional synthetic HsdS_c_ and HsdS_p_ DNA sites, using the plasmid pRL814, which has one naturally occurring HsdS_c_ target site in *lacI* (Fig. 3B). Adding a second HsdS_c_ site to pRL814 (pPK15627) decreased the conjugation efficiency in the parent strain 12.6-fold, whereas adding a HsdS_p_ site to pRL814 (pPK15623) decreased conjugation 1,950-fold. Further, the conjugation of a plasmid bearing two HsdS_p_ target sites (pPK15625) could not be detected even at a frequency 10,000-fold below the conjugation frequency of the plasmid pRL814. Thus, each RM system is a barrier to efficient DNA uptake, and the newly discovered HsdRMS_p_ seems to have the largest impact. Comparing the conjugation efficiency of pPK15627, which has two HsdS_c_ target sites, to that of pPK15623, which has one HsdS_c_ and one HsdS_p_ target site, in strains lacking HsdS_p_ (Δ*hsdS_p_*) or the plasmid-encoded restriction enzyme, HsdR_p_ (Δ*hsdR_p_*), showed that the conjugation efficiency improved only for the plasmid with an HsdS_p_ target site (Fig. 3B). These results indicate that the HsdR_p_ endonuclease does not impact the HsdMS_c_ system. Also, the lack of HsdR_p_ imparts an equivalent mutant phenotype as HsdS_p_, a property expected for proteins that function as a complex.

### Improvement of efficiency of conjugation of foreign genes into Z. mobilis ZM4 requires removal of *hsdS_c_* and *hsdS_p_*.

**FIG 4:**
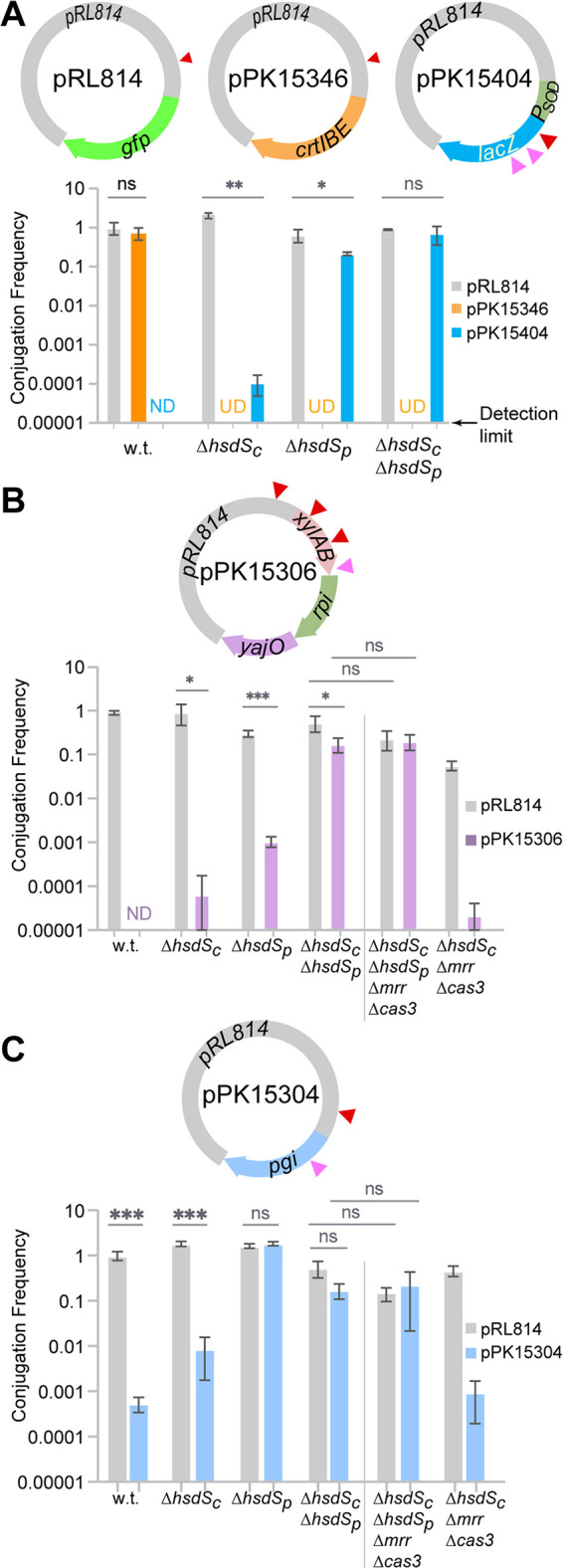
Role of HsdS_c_, HsdS_p_, Mrr, and Cas3 in restricting pPK15404, pPK15306, and pPK15304. (A) The frequency of conjugation of pRL814 into Z. mobilis ZM4 (0.78× 10^−3^ ± 0.09 × 10^−3^) served as a normalization factor for comparing the conjugation frequency (*y* axis) of the other plasmids in wild-type (w.t.) and mutant ZM4 strains. (B) The frequency of conjugation of pRL814 into Z. mobilis ZM4 (0.24 × 10^−3^ ± 0.08 × 10^−3^) served as a normalization factor for comparing the conjugation frequency (*y* axis) of plasmids in wild-type (w.t.) and mutant ZM4 strains as indicated. (C) The frequency of conjugation of pRL814 into Z. mobilis ZM4 (6 × 10^−3^ ± 0.6 × 10^−3^) served as a normalization factor for comparing the conjugation frequency (*y* axis) of plasmids in wild-type (w.t.) and mutant ZM4 strains. When conjugation of a plasmid was below the limit of detection (0.00001), the sample is marked “ND” (not detected). When conjugation experiment of a plasmid was not done, it is marked “UD” (undetermined). Error bars represent the standard deviations of the conjugation frequency means obtained from three independent experiments. Statistical significance was determined using a paired Student's *t* test (*, *P* < 0.05; **, *P* ≤ 0.01; ***, *P* ≤ 0.001; ns, not significant). On the plasmids, red triangles represent restriction sites recognized by HsdS_c_ and pink triangles represent restriction sites recognized by HsdS_p_.

Our goal was to use the knowledge of Z. mobilis ZM4 RM systems to improve uptake of foreign DNA. Therefore, we tested the conjugation efficiency of plasmids containing heterologous DNA of interest into Z. mobilis ZM4 mutants lacking either or both type I RM systems. We utilized pRL814 as the parent plasmid, which has one HsdS_c_ site. As a control for the relative size of the plasmid, we determined the frequency of conjugation into the parent Z. mobilis ZM4 of plasmid pPK15346, which contains the carotenoid-synthesizing genes *crtI*, *crtB*, and *crtE* from Rhodobacter sphaeroides 2.4.1 (35) and which lacks any known restriction sites in addition to the vector site (Fig. 4A). This plasmid mobilized as efficiently as pRL814 into Z. mobilis ZM4, showing that the size of the plasmid did not significantly impact the conjugation efficiency under these conditions.

To evaluate restriction of other foreign genes in Z. mobilis ZM4, we measured the conjugation efficiency of pPK15404, pPK15306, and pPK15304 into Z. mobilis strains (Fig. 4A to C). Plasmid pPK15404 contains E. coli *lacZ*, which serves as a gene expression reporter in many bacteria and contains one HsdS_c_ recognition site and two HsdS_p_ sites. Plasmid pPK15306 contains a synthetic operon composed of E. coli *xylA*, *xylB*, *rpi*, and *yajO*, previously reported to direct xylose into a metabolic pathway (36–38), and contains three HsdS_c_ sites and one HsdS_p_ site. Plasmid pPK15304 contains E. coli *pgi*, involved in conversion of d-glucose 6-phosphate to d-fructose 6-phosphate in glycolysis and contains one recognition site for HsdS_c_ and HsdS_p_. We found that these plasmids were severely restricted in Z. mobilis ZM4, since the conjugation frequency of pPK15404 and pPK15306 was below our detection limit (10^−5^) and the conjugation frequency of pPK15304 was 1,900 times lower than that of plasmid pRL814 (Fig. 4A to C). Deletion of *hsdS_c_* or *hsdS*_p_ alone was not sufficient to overcome the restriction barrier for pPK15306, consistent with both types of target sites present on the plasmid. However, when both systems were deleted, the conjugation frequency was nearly identical to that of the vector control, indicating that the plasmids were no longer restricted. For plasmids pPK15304 and pPK15404, elimination of HsdS_c_ had a small effect, whereas elimination of HsdS_p_ had a much larger effect, since the conjugation frequency was similar to the vector control. These results show that elimination of the HsdMS_c_ and HsdRMS_p_ restriction systems in Z. mobilis ZM4 removes the restriction barrier for multiple sets of foreign genes and enables efficient uptake of heterologous DNA.

### Mrr and Cas3 do not restrict pPK15306 and pPK15304.

Although we do not know the target sequence for Mrr and the target sequence for Cas3 is variably acquired as part of the defense mechanism (13, 14), we created mutants lacking one or both of these activities to determine any contribution to the restriction of foreign DNA by Z. mobilis ZM4. Deletion of either *mrr* or *cas3* alone or in combination did not increase conjugation of pPK15306 or pPK15304 over the limit of detection for this assay (not shown). We also tested the effect of deleting *mrr* and *cas3* in a Δ*hsdS_c_* strain, a background with increased conjugation efficiency, to rule out the possibility that small effects of *mrr* or *cas3* could have been missed in our assay. However, we observed no improvement in conjugation with either plasmid in this strain (Fig. 4B and C). We also tested a strain lacking all four defense activities (Δ*hsdS_c_* Δ*hsdS_p_* Δ*mrr* Δ*cas3* strain) as a recipient for conjugation experiments. This strain was as permissive in conjugating pPK15306 and pPK15304 as the Δ*hsdS_c_* Δ*hsdS_p_* strain (Fig. 4B and C), indicating there is no impairment in conjugation with this quadruple mutant. As a comparison, the doubling times of the wild-type, Δ*hsdS_c_*, Δ*hsdS_c_* Δ*hsdS_p_*, and Δ*hsdS_c_* Δ*hsdS_p_* Δ*mrr* Δ*cas3* strains grown anaerobically in ZRMG medium (see Materials and Methods) at 30°C were 78.5 ± 0.7 min, 97.5 ± 3.5 min, 82.0 ± 2.8 min, and 95.5 ± 0.7 min, respectively. Although Mrr and Cas3 did not impact conjugation efficiency in our experiments, this quadruple mutant strain is available as a potential all-purpose recipient for engineering foreign DNA into Z. mobilis ZM4 in the future, because it would not require any prior knowledge of specific restriction sites in the foreign DNA.

## DISCUSSION

RM systems provide a formidable barrier to entry of foreign DNA (8, 25, 39) and hinder genetic engineering. A thorough analysis of RM systems is a prerequisite to developing genetically tractable strains to promote DNA uptake by either conjugation or transformation. This study reports a comprehensive analysis of restriction systems of Z. mobilis ZM4 and the successful development of strains devoid of multiple restriction activities. A key advance was the discovery of a type I restriction system encoded in native plasmid pZM32 and its target site, which imparts a robust restriction barrier in Z. mobilis ZM4 but is not present in several other Z. mobilis strains.

### A second type I RM system is encoded on a Z. mobilis ZM4 plasmid.

Our bioinformatic analysis confirmed the presence of previously known genes encoding subunits of chromosomally encoded HsdMS_c_ system (21, 22, 25, 40), Mrr, a type IV restriction enzyme (21, 22, 25, 40), and the endonuclease, Cas3, of the type I F CRISPR-Cas system (7, 14). In addition, we also found previously unknown and unannotated genes for a complete type I RM system (*hsdRMS_p_*) on plasmid pZM32. The proteins of this type I RM system (HsdRMS_p_) are very distinct from the proteins of the previously annotated chromosomal type I RM system (HsdMS_c_); the HsdS_c_-HsdS_p_ proteins and the HsdM_c_-HsdM_p_ proteins share only 34% and 28% sequence similarity, respectively.

In this work, we showed that removal of both type I RM systems improved conjugation efficiency of heterologous genes in Z. mobilis ZM4. However, the most formidable barrier to improving conjugation efficiency was the newly identified plasmid encoded type I restriction system. As reported previously (22, 25) and confirmed here, elimination of the activity encoded by the HsdMS_c_ system provided a small improvement in uptake of plasmids containing heterologous DNA. However, since most of the plasmids used in our studies had recognition sites for both the *hsdMS_c_* and the *hsdRMS_p_* systems, an optimal improvement in conjugation efficiency was observed when both activities were eliminated. Z. mobilis mutants lacking *mrr* have previously been shown to increase DNA uptake of the shuttle vector pBBR1MCS-3 (22), although we did not observe any increase with the plasmids used in our experiments. Since the recognition sequence for Mrr in Z. mobilis is unknown, it is possible that the plasmids used in this study lacked the sequence for Mrr restriction. Nevertheless, because future synthetic biology plasmid designs may have a target site for Mrr or the CRISPR-Cas3 system, we created a quadruple mutant strain (Δ*hsdS_c_* Δ*hsdS_p_* Δ*mrr* Δ*cas3*; PK15509) lacking all four defense systems of Z. mobilis as a suitable all-purpose platform strain for future genetic engineering of Z. mobilis ZM4.

An important advance was the finding that the restriction activity of the HsdMS_c_ system in Z. mobilis ZM4 is less than that of the HsdRSM_p_ system but that HsdMS_c_ is still an active RM system that needs to be evaded for maximal DNA uptake. As reported previously (25) and confirmed here, no gene encoding a full-length HsdR_c_ could be found in Z. mobilis ZM4. Since other bacteria are known to share a single HsdR with multiple HsdMS systems (6, 41, 42), we assessed if HsdR_p_ is shared with HsdMS_c_ system in Z. mobilis ZM4. Because elimination of HsdR did not affect the conjugation efficiency of a plasmid that is generally restricted by the HsdMS_c_ system, this result suggests that HsdR_p_ is not shared with the HsdMS_c_ system in Z. mobilis ZM4. Rather, we found that HsdM_c_ contains a conserved domain found in the N terminus of type I restriction enzyme R protein (HsdR_N). Recently, it has been proposed that such proteins evolved from a fusion of the N terminus of HsdR with HsdM of type I RM system (29). This class of fusion protein is proposed to retain the methylation activity but lack the motor and helicase domains responsible for reeling in DNA sequence that promote ATP-driven cleavage, suggesting that DNA cleavage occurs in closer proximity to the HsdS binding site than with a typical type I HsdR (29).

We have also demonstrated the usefulness of our previously published markerless genome modification method (5) to generate restriction deficient strains by sequentially deleting the four genes encoding the different defense systems from different regions of the genome without introduction of any permanent antibiotic resistance markers. Thus, the absence of any antibiotic resistance cassettes provides an excellent starting point for metabolic strain engineering. Further, the restriction systems can easily be reintroduced back into engineered strains if needed for industrial robustness. We also found that deletion of genes from one of the endogenous plasmids showed the same frequency as deletion of genes from chromosome. Thus, this method can be used for further modification of the endogenous plasmids.

### Z. mobilis ZM4 ZMO1005 possibly encodes a CcrM-like methyltransferase.

In addition to methylome sequencing analysis enabling identification of the target sequence for the plasmid-encoded type I RM system and confirming the sequence of the chromosomally encoded system (8, 43), we also found an additional methylated sequence indicative of the methyltransferase CcrM, which methylates the adenine of GANTC in alphaproteobacteria (44–46).

Bioinformatic analysis indicated that ZMO1005 encodes a protein with an *N*^6^-methyltransferase family domain and showed 60% similarity with CcrM, a class β-methyltransferase of *Caulobacter* sp. Additionally, ZMO1005 contains conserved motif IV (DLIFADPPYNLQLGG) and motif I (ILDPFFGVGTTGAAA) of class β-methyltransferases (33). *ccrM* is prevalent in alphaproteobacteria and plays a vital role in DNA replication, DNA repair, and gene regulation (32, 43, 47, 48). CcrM, DnaA, GcrA, CtrA, and SciP are five master regulators that together control cell cycle progression by modulating epigenetic changes (43, 46–48). BLASTP analysis of these proteins against Z. mobilis ZM4 genome indicates the presence of all except *sciP* in Z. mobilis ZM4. Overall, this analysis indicates that CcrM (ZMO1005) might play an important role in Z. mobilis ZM4, which needs to be explored.

### Future prospect of genome engineering in Z. mobilis ZM4.

A primary focus of this work was to identify RM systems of Z. mobilis ZM4 and to create strains with improved ability to take up plasmids encoding heterologous metabolic functions. Indeed, we improved the conjugation efficiency of plasmids containing genes that encode β-galactosidase, a xylose utilization pathway (36–38), and phosphoglucoisomerase (49, 50). Given the utility of β-galactosidase as a widespread reporter in gene expression studies, our development of a strain lacking RM systems provides a new useful tool for analyzing promoter-specific gene expression in Z. mobilis ZM4. Similarly, the efficient introduction into Z. mobilis ZM4 of heterologous metabolic functions like xylose utilization (36) or phosphoglucoisomerase (49) provides a proof of concept for the usefulness of these strains for future metabolic engineering.

### Conclusion.

This study illustrates the impact of Z. mobilis ZM4 RM systems on restricting foreign DNA. We conducted comprehensive bioinformatic, genetic, and high-throughput methylome sequence analyses to identify all RM systems of Z. mobilis ZM4. We created a strain lacking all restriction systems, which accepts all foreign genes tested so far. This work will therefore help accelerate genetic engineering of Z. mobilis ZM4 by eliminating restriction of heterologous DNA and improving DNA uptake efficiency.

## MATERIALS AND METHODS

### Materials.

All restriction endonucleases, Q5 polymerase, and Gibson assembly HiFi master mix were from New England Biolabs, Inc. GOTaq Flexi DNA polymerase was from Promega. Primers were obtained from Integrated DNA Technologies (IDT). A Sony MA900 fluorescence activated cell sorter (FACS) was used for sorting nonfluorescent cells from fluorescent cells, and an Azure C600 imager was used for screening of fluorescent colonies as previously described (5).

### Strains, plasmids, and growth conditions.

Bacterial strains, primers, and plasmids used in this study are listed in Tables 2 to 4, respectively. Z. mobilis ZM4 and its derivatives were grown in ZRMG medium (1% yeast extract, 0.2% KH_2_PO_4_, 2% glucose) (5), and E. coli strains were grown in Luria-Bertani (LB) medium (51). Chloramphenicol was used at a final concentration of 120 μg/ml for Z. mobilis ZM4 and 20 μg/ml for E. coli strains. Spectinomycin was used at a final concentration of 120 μg/ml for Z. mobilis ZM4 and 50 μg/ml for E. coli strains. For growth of E. coli WM6026, 0.1 mM *m*-diaminopimelate (DAP) was added to liquid media and 0.15 mM DAP to solid media (5).

**TABLE 2:** Strains

Strain	Description	Source and/or reference
DH5α	E. coli F^−^ *endA1 glnV44 thi*-*1 recA1 relA1 gyrA96 deoR nupG purB20* ϕ80d*lacZ*ΔM15 Δ (*lacZYA*-*argF*)*U169 hsdR17*(r_K_^−^ m_K_^+^) λ^−^	Lab collection
WM6026	E. coli*lacI*^q^*rrnB3* Δ*lacZ4787 hsdR514* Δ*araBAD567* Δ*rhaBAD568 rph*-*1 att*λ::pAE12(Δ*ori*R6K-*cat*::Frt5) Δ*endA*::Frt *uidA*(ΔMluI)::*pir att*HK::pJK1006 (Δ*ori*R6K-*cat*::Frt5; *trfA*::Frt) Δ*dapA*::Frt	67
Z. mobilis ZM4 (PK15256)	Zymomonas mobilis ZM4 ATCC 31281	GLBRC (31)^*a*^
PK15394	Z. mobilis Δ*cas3* (*zmo0681*)	This work
PK15395	Z. mobilis Δ*cas3* (*zmo0681*) Δ*mrr* (*zmo0028*)	This work
PK15407	Z. mobilis Δ*cas3* (*zmo0681*) Δ*mrr* (*zmo0028*) Δ*hsdS_c_* (*zmo1933*)	This work
PK15410	Z. mobilis Δ*hsdS_c_* (*zmo1933*)	This work
PK15509	Z. mobilis Δ*cas3* (*zmo0681*) Δ*mrr* (*zmo0028*) Δ*hsdS_c_* (*zmo1933*) Δ*hsdS_p_* (*zmop32x028*)	This work
PK15510	Z. mobilis Δ*cas3* (*zmo0681*) Δ*mrr* (*zmo0028*) Δ*hsdR_p_* (*zmop32x025*)	This work
PK15527	Z. mobilis Δ*hsdS_c_* (*zmo1933*) Δ*hsdS_p_* (z*mop32x028*)	This work

a GLBRC, Great Lakes Bioenergy Research Center.

**TABLE 3:** Primers

Plasmid constructed	Plasmid/gene amplified	Primer	Sequence (5′–3′)
pPK15436 (pRL814-*crtIEB*)	pRL814 backbone	P1	ATGTATATCTCCTTCTTAAAGTTAAAC
		P2	GCTTGATATCGAATTCCTG
	*crtBI* of R. sphaeroides	P3	TTTAAGAAGGAGATATACATATGCCCTCGATCTCGCCC
		P4	TATTTATAATAGAAAGTAAAGACTAGATCGGGTTGGCCCG
	*crtE* of R. sphaeroides	P5	CCCGATCTAGTCTTTACTTTCTATTATAAATAAAGGAGACCTTTCATGAGGCACAAGATGGCGTTTGAACAGC
		P6	GCAGGAATTCGATATCAAGCTCAGACGCGGGCCGCGAC
pPK15404 (pRL814-*lacZ*)	pRL814 backbone	P7	CTTAAGGCCGGATCTTGCGGCCCC
		P8	TGGGATTACACATGGCATG
	Promoter of *sod* of Z. mobilis	P9	GAGGGGCCGCAAGATCCGGCCTTAAGGCTGGGAATAGCATTTCTC
		P10	TCATGGTCATGATGTGATCTCCCAATTC
	*lacZ* of E. coli MG1655	P11	AGATCACATCATGACCATGATTACGGATTCAC
		P12	CCATGCCATGTGTAATCCCAGCGAAATACGGGCAGACATG
pPK15306 (pRL814-*xylA*, *xylB*, *rpi* and *yajO*)	pRL814 backbone	P13	AATTTAGTATATCCTTTGTCGGGTAATTTTTTAATAATTGTTATCCGCTCACAATTG
		P14	TGGGATTACACATGGCATG
	*xylA* of E. coli MG1655	P15	ATTCAATTGTGAGCGGATAACAATTATTAAAAAATTACCCGACAAAGGATATACTAAATTATGCAAGCCTATTTTGACC
		P16	TTCTGTTAGTCTGCTTTGTTATTTGTCGAACAGATAATGG
		P17	ATTCAATTGTGAGCGGATAAC
	*xylB* of E. coli MG1655	P18	GTTCGACAAATAACAAAGCAGACTAACAGAAGGAAGTAACACATGTATATCGGGATAGATCTTG
		P19	TTTCCTTCGTTTGCCCTTTACGCCATTAATGGCAG
		P20	GTTCGACAAATAACAAAGCAGAC
	*rpi* of E. coli MG1655	P21	ATTAATGGCGTAAAGGGCAAACGAAGGAAACGACAGGAAGTACATATATGACGCAGGATGAATTG
		P22	GATTATAAAGTTAATTTGCGTCATTTCACAATGGTTTTGAC
		P23	ATTAATGGCGTAAAGGGC
	*yajO* of E. coli MG1655	P24	CATTGTGAAATGACGCAAATTAACTTTATAATCAAGGAACAGAAACAGATGCAATACAACCCCTTAG
		P25	CTCATCCATGCCATGTGTAATCCCATTATTTAAATCCTACGACAGGATG
		P26	CATTGTGAAATGACGCA
pPK15304 (pRL814-*pgi*)	pRL814 backbone	P27	AATTGTTATCCGCTCACAATTG
		P28	TGGGATTACACATGGCATG
	*pgi* of E. coli MG1655	P29	ATTGTGAGCGGATAACAATTAAAGTCACAATTCTCAAAATCAGAAG
		P30	CCATGCCATGTGTAATCCCACGATGATTAACCGCGCCA
pPK15303-2	pPK15303 backbone	P31	CGGCCTGTTTAGCGCTCGGTCTTGCCTT
		P32	GGGCATAAACGTGCCGAGGATGACGATG
	Upstream of *mrr* of Z. mobilis	P33	TCCTCGGCACGTTTATGCCCGTGACAAC
		P34	TTCAAAAAGCTATCGAGCCTTTTACTTAAAATAATC
	Downstream of *mrr* of Z. mobilis	P35	AGGCTCGATAGCTTTTTGAAAAAGCCGTTTC
		P36	ACCGAGCGCTAAACAGGCCGCTAACCTAG
pPK15357	pPK15303-2 with modified RBS of *gfp*	P37	AAGATATTAGAAGGAGGTCAAAAATGAGCAAAGGAGAAGAACT
		P38	TTTTGACCTCCTTCTAATATCTTGTTAAACTAATTCTAGATGT
pPK15381	pPK15357 backbone	P39	GTGCCGAGGATGACGATG
		P40	AGCGCTCGGTCTTGCCTT
	Upstream of *hsdSc* of Z. mobilis	P41	CTCATCGTCATCCTCGGCACTTCATTGAGCGCAATCTG
		P42	GGTCAAGGTACAGATCGCTTAGGCGATC
	Downstream of *hsdS_c_* of Z. mobilis	P43	AAGCGATCTGTACCTTGACCATGCAGCTG
		P44	GCAAGGCAAGACCGAGCGCTTTGTGCCTGCACATGCTG
pPK15472	Upstream of *hsdS_p_* of Z. mobilis	P45	CTCATCGTCATCCTCGGCACGACGATGGAGCAAAGACAG
		P46	TAGTGCTCATTATTCCCTCAGTCCATTAGAC
	Downstream of *hsdS_p_* of Z. mobilis	P47	TGAGGGAATAATGAGCACTACTACCGATATCGTTG
		P48	GCAAGGCAAGACCGAGCGCTATCACTTCCCCCGCCTGAG
pPK15474	Upstream of *hsdR*_p_ of Z. mobilis	P49	CTCATCGTCATCCTCGGCACATGCGGGCGAACATGCCC
		P50	TGGCTATGGGATGCGTGCCAACGACCCC
	Downstream of *hsdR_p_* of Z. mobilis	P51	TGGCACGCATCCCATAGCCATTTCCGATTAC
		P52	GCAAGGCAAGACCGAGCGCTACGATCAGTTGCTCGAAAAAG
pPK15380	Upstream of *cas3* of Z. mobilis	P53	TGCATTATGATTTATTCAGAAATGATGGAAAATATTATGCATC
		P54	GATCTTCTCCCCGCAACCAATAA
	Downstream of *cas3* of Z. mobilis	P55	TCTGAATAAATCATAATGCAGGCTCGGC
		P56	CTCATCGTCATCCTCGGCACCGTTTATCGGCTTTGCTC
pPK15536	pRL814 with HsdS_p_ target sequence	P57	GAGTTCCCCCGGGGGATCCCTAGTTCT
		P58	GCGCTTCCTGCAGGAATTCGATATCAAGCT

**TABLE 4:** Plasmids

Plasmid	Description^*a*^	Source and/or reference
pRL814	Broad-host-range plasmid containing pBBR-1 origin of replication, *lacI*^q^ gene, P_T7A1-O34_-*gfp*, *mob*, and spectinomycin resistance cassette	GLBRC (52)^*b*^
pPK15305	pRL814 with *pgi* gene cassette replacing the *gfp* locus	This work
pPK15306	pRL814 with *xylAB*, *rpi*, and *yajO* gene cassette inserted at the *gfp* locus	This work
pPK15346	pRL814 with *crtIEB* cassette inserted at the *gfp* locus	This work
pPK15404	pRL814 with *lacZ* cassette inserted at the *gfp* locus	This work
pPK15303	Suicide plasmid; *cat gfp* p15A *ori mob* with 500-bp regions upstream and downstream of the *ldh* gene	5
pPK15303-2	Suicide plasmid pPK15296 with 500-bp regions upstream and downstream of the *mrr* gene in place of *ldh* flanking regions	This work
pPK15343	pPK15303-2 with optimized RBS for *gfp*	This work
pPK15357	pPK15303-2 with optimized RBS for enhanced expression of *gfp*	This work
pPK15380	Suicide plasmid pPK15357 with 500-bp regions upstream and downstream of the *cas3* gene in place of *mrr* flanking regions	This work
pPK15381	Suicide plasmid pPK15357 with 500-bp regions upstream and downstream of the *hsdS_c_* gene in place of *mrr* flanking regions	This work
pPK15472	Suicide plasmid pPK15357 with 500-bp regions upstream and downstream of the *hsdS_p_* gene in place of *mrr* flanking regions	This work
pPK15474	Suicide plasmid with 500-bp regions upstream and downstream of the *hsdR_p_* gene	This work
pPK15536	pRL814 with target sequence of HsdS_p_ (5′CGAAGCGCGAGTTCC3′) at the SmaI restriction site	This work
pPK15617	pRL814 lacking HsdS_c_ target sequence (5′GTCAGTGGGCTG3′; nt 364 to 375 from the translational start site of *lacI*)	This work
pPK15619	pPK15617 with HsdS_p_ target sequence (5′CGAAGCGCGAGTTCC3′) at the SmaI restriction site	This work
pPK15621	pPK15617 with HsdS_c_ target sequence (5′GTCAGTGGGCTG3′) at the SmaI restriction site	This work
pPK15623	pRL814 with HsdS_p_ target sequence (5′CGAAGCGCGAGTTCC3′) at the SmaI restriction site	This work
pPK15625	pRL814 with two HsdS_p_ target sequences, the first in *lacI* replacing HsdS_c_ target sequence (5′CAGCCCACTGAC-3′) with the HsdS_p_ target sequence (5′CGAAGCGCGAGTTCC3′) and the second at the SmaI restriction site (insertion of 5′CGAAGCGCGAGTTCC3′)	This work
pPK15627	pRL814 with HsdS_c_ target sequence (5′GTCAGTGGGCTG3′) at the SmaI restriction site	This work

a RBS, ribosome binding site.

b GLBRC, Great Lakes Bioenergy Research Center.

### Sample preparation for SMRT sequencing.

Cell lysis and genomic DNA isolation were done as described by using a MasterPure complete DNA and RNA extraction kit (Lucigen) and PacBio single-molecule real-time (SMRT) DNA sequencing. Strains were grown anaerobically in 50 ml ZRMG until late exponential phase and harvested by centrifugation at 6,000 × *g* at 4°C. For cell lysis, the cell pellet was resuspended in 10 ml TE buffer (10 mM Tris-HCl, 1 mM EDTA [pH 8]), incubated with 5 μl lysozyme (30,000 U/μl; Lucigen) at 37°C and 30 min, followed by the addition of 100 μl tissue and cell lysis solution (Lucigen) premixed with 20 μl 10 mg/ml proteinase K and then incubated at 65°C for 30 min. High-molecular-weight genomic DNA was isolated from the lysed cell suspension by extracting (once or twice) with an equal volume of phenol-chloroform-isoamyl alcohol (24:24:1) and centrifuging at 13,000 × *g* at 4°C for 10 min to separate the phases. The aqueous phase was then extracted with an equal volume of chloroform-isoamyl alcohol (24:1) as described above and transferred to a tube containing ammonium acetate (to a final concentration of 0.75 M) and 2.5 volumes of absolute ethanol. Genomic DNA was spooled out and resuspended in 200 μl TE buffer. Thirty micrograms of DNA was used for SMRT DNA sequencing at the Institute for Genome Sciences, University of Maryland School of Medicine, Baltimore, MD.

### Conjugation of plasmids into Z. mobilis ZM4.

A DAP auxotrophic donor, E. coli WM6026, was used to conjugate plasmids into Z. mobilis ZM4 or its mutant derivatives as described previously (5). A stable vector pRL814 or its derivatives, which contains a broad-host-range MobV type conjugation system and pBBR-1 origin of replication, were used for conjugation. CFU were determined after conjugation by plating 100 μl of appropriate 10-fold dilutions of conjugation mixtures onto ZRMG plates, with or without antibiotic. The conjugation frequency was determined by dividing the CFU measured from plates with antibiotic (total number of exconjugants) by the CFU from plates without antibiotic (total number of viable cells). The conjugation frequency from three independent experiments was used to determine the means and standard deviations. Conjugation frequency was determined by normalizing the frequency of one plasmid to that of a control plasmid. The limit of detection in this assay is a conjugation efficiency of 0.00001. GraphPad Prism was used to calculate means, standard deviations, and *P* values.

### Construction of plasmids with the HsdMS_c_ and HsdRMS_p_ target sites.

pRL814-derived plasmids containing no, single, or multiple HsdMS_c_ and HsdRMS_p_ target sites were constructed at Genewiz, Inc., using site-directed mutagenesis as follows: (i) pPK15617 contains a deletion of the HsdS_c_ recognition sequence (5′GTCAGTGGGCTG3′; nucleotides [nt] 364 to 375 from the translational start site of the *lacI* gene) from pRL814; (ii) pPK15621 contains an insertion of the HsdS_c_ recognition sequence (5′GTCAGTGGGCTG3′) into pPK15617 at the SmaI restriction site; (iii) pPK15619 contains an insertion of the HsdS_p_ recognition sequence (5′CGAAGCGCGAGTTCC3′) into pPK15617 at the SmaI restriction site; (iv) pPK15627 contains an insertion of a second HsdS_c_ recognition sequence (5′GTCAGTGGGCTG3′) into pRL814 at the SmaI restriction site; (v) pPK15623 contains an insertion of the HsdS_p_ recognition sequence (5′CGAAGCGCGAGTTCC3′) into pRL814 at the SmaI restriction site; and (vi) pPK15625 contains two HsdS_p_ recognition sequences (5′CGAAGCGCGAGTTCC3′) in pRL814; one replaces the HsdS_c_ recognition sequence (5′GTCAGTGGGCTG3′) in *lacI*, and the second is an insertion at the SmaI restriction site. The sequence of the conjugation mobilization element (*mobA*) and the regions of insertions or deletions of the HsdMS_c_ and HsdRMS_p_ target sites were confirmed by Sanger sequencing.

### Cloning of heterologous genes into vector pRL814.

Cloning of heterologous genes into vector pRL814 (52) was achieved by PCR amplification of the vector and indicated genes followed by Gibson assembly using NEBuilder HiFi DNA assembly cloning kit. The Gibson assembly products were transformed into E. coli DH5α using a heat shock method (53). Plasmids were isolated using a plasmid extraction kit (Thermo Fisher Scientific) and confirmed by PCR and Sanger sequencing using primers specific to the DNA fragment junctions. DNA fragments were amplified using primers described in Table 3 for the plasmids listed in Table 4.

### Deletion of genes.

Deletion of genes from the Z. mobilis ZM4 genome relied on a homologous recombination method (5) where in the first step, a suicide plasmid, pPK15534 (Table 4), containing 500 bp of DNA upstream and downstream of the target gene to be deleted was conjugated into the recipient Z. mobilis strain from the donor E. coli DAP auxotroph WM6026. Exconjugants that contained plasmid DNA recombined at either the upstream or the downstream position of the gene were selected on ZRMG solid medium containing 120 μg/ml chloramphenicol. The recombinant strains were grown without selection and sorted by FACS to enrich for nonfluorescent strains that had lost the plasmid. The desired deletion strain was identified by PCR assays with specific primers, and the deletion boundaries were confirmed by Sanger sequencing. pPK15357 was used to delete *mrr*, pPK15380 was used to delete *cas3*, pPK15381 was used to delete *hsdS_c_*, pPK15472 was used to delete *hsdS_p_*, and pPK15474 was used to delete *hsdR_p_* (Table 4).

### Identifying conserved domains of restriction systems.

Z. mobilis ZM4 open reading frames (ORFs) (31) were searched for conserved domains using CDD-Search at NCBI (34, 54), which uses the CDD database or the SRA (Sequence Read Archive) database (55) to scan for a set of precalculated position-specific scoring matrices (PSSM; a unique identifier for domain) in a peptide. CDD-search uses RPS-blast, a variant of PSI-blast, to translate DNA sequence into peptide sequences for all six frames and performs a scan for a set of PSSM with each peptide sequence. These domains included the specificity-determining (HsdS) activity of the RM Type1_S-TRD-CR-like superfamily (30, 34); methyltransferase (HsdM) of the *N*^6^-methyltransferase superfamily (34, 56); the type I restriction endonuclease (HsdR) superfamily (34, 57); type IV restriction endonuclease activities of the Mrr type endonuclease superfamily and PD-(D/E)XK superfamily (58, 59); type II restriction endonuclease activities of the GIY-YIG superfamily (58, 60) and the HNH endonuclease superfamily (58, 61); and signature nucleases of CRISPR-Cas system-associated endonucleases, such as Cas3, Cas9, and Cas10 (13, 62). The complete genome sequence, GenBank entry GCA_003054575.1, was analyzed in segments of 8 to 10 kb as described for systematic screening for modification-dependent restriction endonucleases in E. coli (63). The CDD identifiers were then analyzed for domains using phmmer, InterPro, and UniProt databases (64).

### Bioinformatic analysis of *hsdMS_c_* and *hsdRMS_p_* from different Z. mobilis strains.

To screen for *hsdMS_c_* and *hsdRMS_p_* in different Z. mobilis strains, a local tBLASTn search (65) function for *hsdS_c_*, *hsdM_c_*, *hsdS_p_*, *hsdM_p_*, and *hsdR_p_* of Z. mobilis strain ZM4 against 16 unique genome sequence assemblies obtained from the NCBI database was performed. Sequence comparison of *hsdS_c_* and *hsdS_p_* of Z. mobilis ZM4 was done using NCBI BLAST and Clustal Omega (66).
